# Clinical Management of Hypodontia of Two Mandibular Incisors

**DOI:** 10.1155/2021/6625270

**Published:** 2021-02-03

**Authors:** Sergio Paduano, Lorenza Barbara, Domenico Aiello, Marianna Pellegrino, Felice Festa

**Affiliations:** ^1^Department of Health, University “Magna Graecia” of Catanzaro, Viale Europa, Loc. Germaneto, 88100 Catanzaro, Italy; ^2^Private Practice, Via G. A. Acquaviva 37, 81100 Caserta, Italy; ^3^Department of Oral Medical Sciences and Biotechnology, University “G. d'Annunzio” of Chieti-Pescara, Via dei Vestini, 32 66013 Chieti, Italy

## Abstract

This article presents the clinical management of a patient with bilateral congenitally missing mandibular incisors. This condition is relatively rare and always needs a careful diagnosis and treatment planning. The chosen treatment strategy for this patient included space closure by protraction of the mandibular posterior teeth and canine substitution of missing incisors. Furthermore, the problems arising from this treatment plan, such as premolar-protected occlusion and tooth size discrepancy, are discussed. From the case presented in this study, we can conclude that space closure may be considered an efficient treatment approach for achieving satisfactory functional and aesthetic results.

## 1. Introduction

Hypodontia is defined as the congenital absence of one or a few teeth [1]. Oligodontia and anodontia are more severe forms of dental agenesis, characterized by the absence of more than six teeth and by the complete absence of teeth, respectively. These forms are usually associated with other systemic conditions such as Down syndrome, ectodermal dysplasias, and Ellis-van Creveld syndrome [1, 2]. Four main theories have been reported about the etiology of dental agenesis; it might be considered an expression of the evolutionary trend or it might be due to environmental or systemic factors such as trauma, inflammation, infections in the jaw, or disturbance of the endocrine system [3]. Heredity or familial distribution can be the primary cause. In addition to the hypodontia of lower incisors, anomalies in the development of the mandibular symphysis may affect the formation of tooth buds [3]. Hypodontia of mandibular lateral and central incisors is relatively rare; in fact, with a third molar exclusion, prevalence ranges about 6.1% for the central and 4.3% for the lateral of both congenitally missing teeth in the mondial population [4]. A higher prevalence can be found in the Japanese, Chinese, and Korean population [5, 6]. According to Polder et al., the mandibular second premolars are the most frequently missing teeth, followed by the maxillary lateral incisors and maxillary second premolars; furthermore, the prevalence of hypodontia can range from 3 to 6.3% and is higher in females than in males [7, 8]. Several previous studies have reported on the congenital absence of permanent mandibular incisors and mandibular symphysis morphology [9–12]. Buschang et al. found that vertical and horizontal growth changes during childhood and puberty are most pronounced in the upper half of the mandibular symphysis and that tooth eruption plays a critical role in the continuous growth of the mandibular symphysis, resulting in an increase in the height of the mandibular body [9]. Moreover, Endo et al. reported that patients with congenital absence of permanent mandibular incisors exhibit a smaller mandibular symphysis area and a greater retroclination of mandibular alveolar bone than patients without hypodontia; they concluded that these findings should be taken into consideration in planning orthodontic treatment on patients with agenesis of these teeth [10]. G. V. Newman and R. A. Newman described three treatment strategies for patients with hypodontia of lower incisors: space closure with protraction of the mandibular canines and posterior teeth forward, extraction of maxillary premolars to balance the missing mandibular incisors, and space opening for a fixed denture or implant-supported restorations [13]. The choice between space opening and closure depends upon several parameters, including age of the patient, facial typology and profile, and occlusal relationship. This article presents the orthodontic management of a patient with bilateral congenitally missing mandibular incisors; the treatment plan included space closure with canine substitution for missing lower incisors.

## 2. Case Presentation

### 2.1. Diagnosis and Treatment Plan

**Figure 1:**
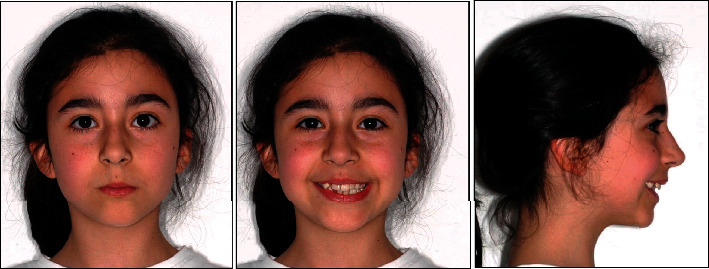
Initial facial photographs.

**Figure 2:**
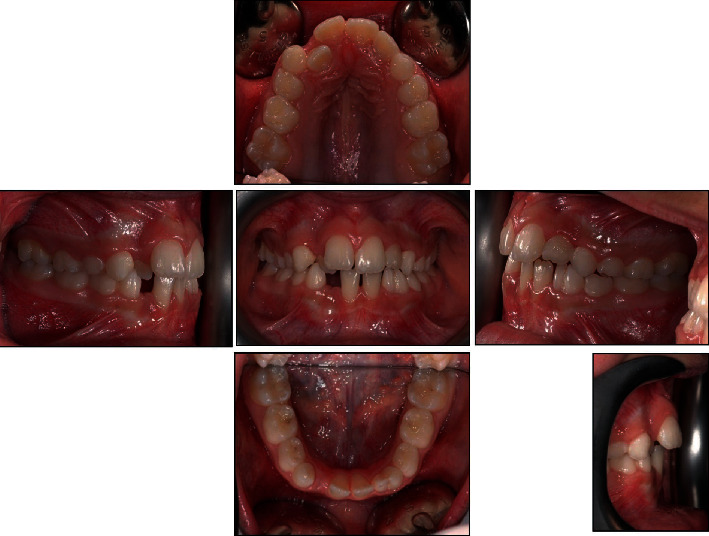
Initial intraoral photographs.

The patient was 10 years old with no significant systemic medical history and no family history of dental anomalies [14]. The initial extraoral and intraoral photographs of the patient are shown in Figures 1 and 2. She presented this objective problem list:

- Missing mandibular lateral incisors
- Crossbite of the maxillary right lateral incisor
- Presence of the deciduous lower lateral incisor
- Wide spacing on the mandibular arch
- Dolichofacial typology
- Skeletal class II
- Class II molar relationship
- Lower dental midline shifted to the left

**Figure 3:**
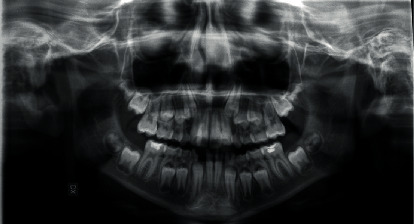
Initial panoramic radiograph.

**Figure 4:**
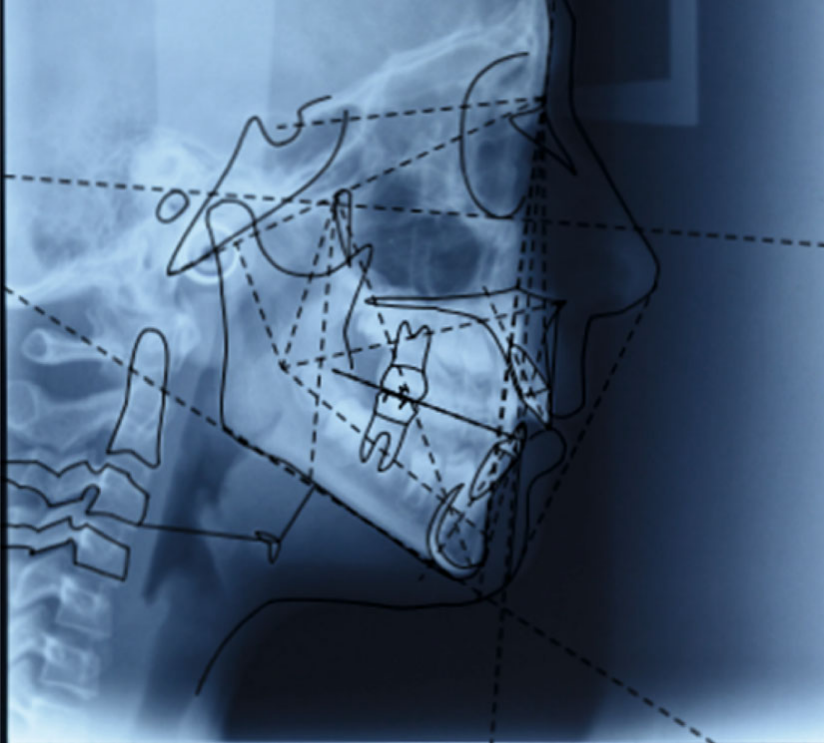
Initial lateral cephalogram.

**Figure 5:**
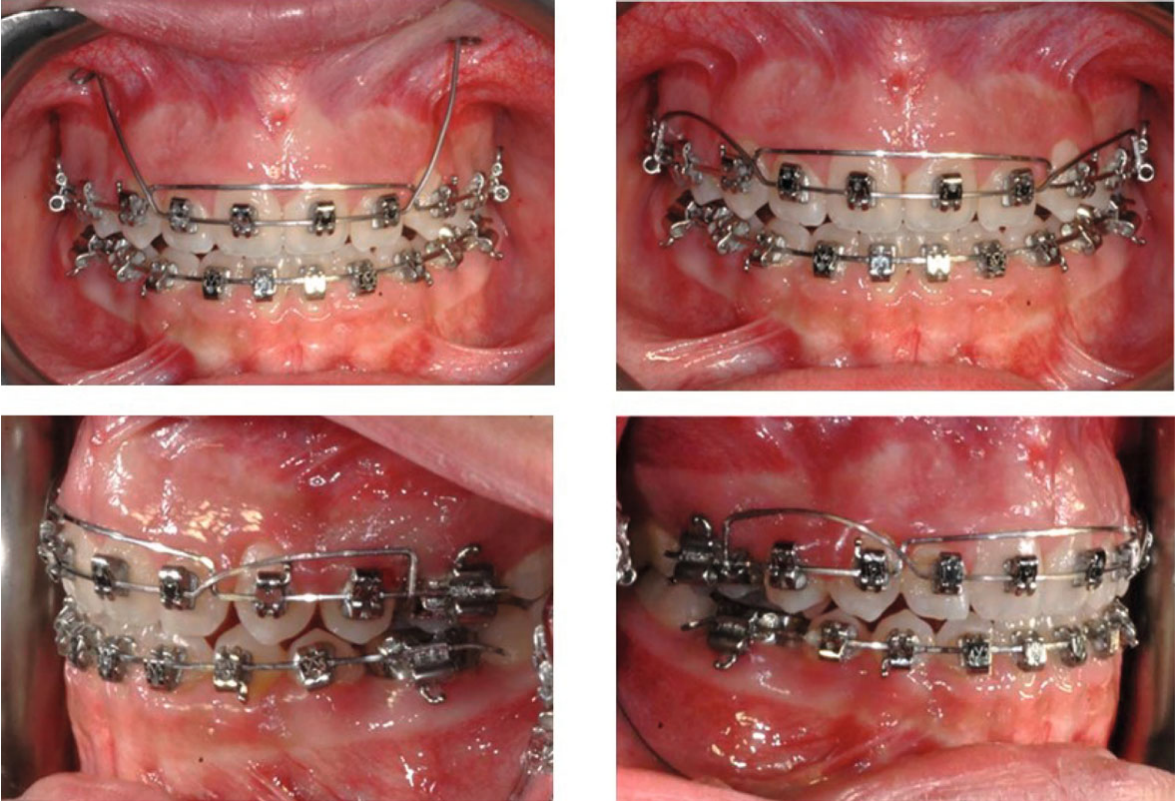
Second-order bend on the SS archwire with a torque arch.

**Figure 6:**
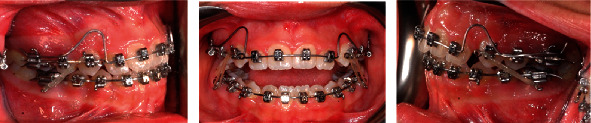
Translation utility arch (TRUA).

The patient did not present signs or symptoms of temporomandibular disorders according to the Research Diagnostic Criteria for Temporomandibular Disorders [15, 16]. A panoramic radiograph and lateral cephalogram at the start of the treatment are shown in Figures 3 and 4. The cephalometric evaluation highlighted a dolichofacial typology with a sagittal skeletal relationship of class II (Table 1). Based on these findings, the chosen treatment plan involved space closure, lower canine to lateral incisor substitutions, and the extraction of the upper first premolars in order to finish the occlusion in class I molar relationship. This treatment strategy was selected according to the age of the patient, facial typology, and requirement of molar correction. When canines are substituted for lateral incisors, their greater mesiodistal width may cause a Bolton discrepancy for the mandibular anterior excess. During the treatment planning, Bolton analysis was performed and it highlighted that proximal stripping on the mandibular teeth was necessary to achieve an ideal overjet and overbite; however, the patient's parent refused this treatment. A fixed self-ligating multibracket appliance Roth prescription, slot size 0.022^″^ × 0.028^″^ (time 2 American Orthodontics, 3524 Washington Avenue, Sheboygan, WI 53081, United States) was placed to align, to level, and to close the space in the mandibular arch. Furthermore, a transpalatal arch was used for the rotational control and for the correction of the upper first molars' torque. The archwire treatment sequence included the following:

- 0.014^″^ HA Ni-Ti upper and lower alignment archwires (Tanzo Wire, American Orthodontics, 3524 Washington Avenue, Sheboygan, WI 53081, United States)
- 0.016^″^ Australian upper and lower archwires (AJ Wilcock Regular Plus, Hay Mills, Birmingham B25 8DW, West Midlands, United Kingdom) for little space closure
- 0.018^″^ × 0.025^″^ HA Ni-Ti upper and lower archwires (Tanzo Wire, American Orthodontics, 3524 Washington Avenue, Sheboygan, WI 53081, United States) for three-dimensional aligning
- Because of insufficient overbite and torque, 0.019^″^ × 0.025^″^ SS upper archwire (GAC, 355 Knickerbocker Avenue, Bohemia, NY 11716, United States) and double cantilever were used. A second-order bend distal to the lateral incisors was modelled on the SS archwire, so that the anterior part of the archwire was placed gingival to the brackets, in order to allow incisor extrusion due to the root palatal torque from the double cantilever. Indeed, the double cantilever 0.017^”^ × 0.025^″^ TMA (1717 West Collins, Orange, CA 92867, United States) generates a moment on the incisors, that is in balance with the moment of the system consisting of the anterior extrusive force and the posterior intrusive force (Figure 5)
- After a retraction of both cuspids, a translation utility arch (TRUA) [17] was used to preserve the obtained torque during the space closure between lateral incisors and canines (Figure 6)
- 0.018^″^ × 0.025^″^ HA Ni-Ti (Tanzo Wire, American Orthodontics, 3524 Washington Avenue, Sheboygan, WI 53081, United States) with bend back was used to solve misalignments and to avoid space reopening
- 0.019^″^ × 0.025^″^ SS archwires (GAC, 355 Knickerbocker Avenue, Bohemia, NY 11716, United States) were used for arch wire coordination and finishing

### 2.2. Treatment Results

**Figure 7:**
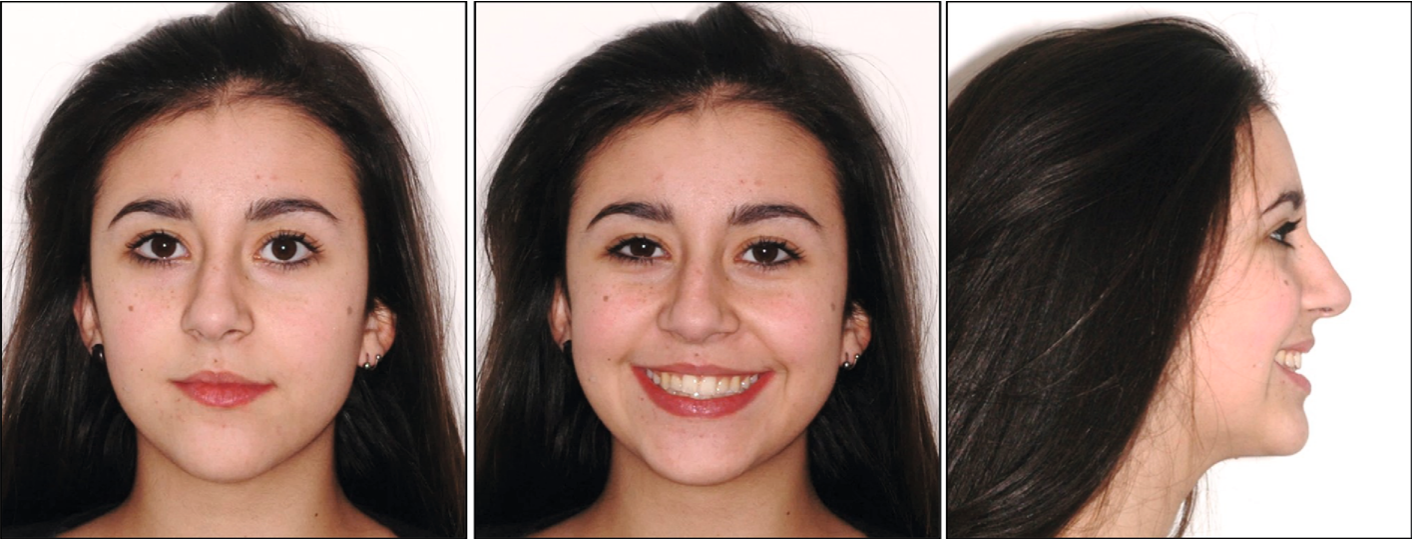
Final facial photographs.

**Figure 8:**
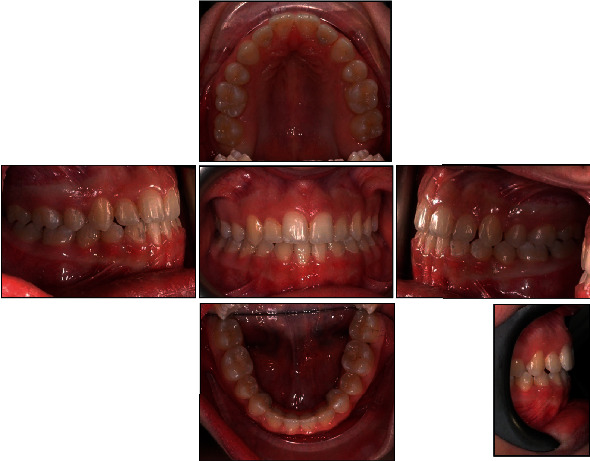
Final intraoral photographs.

**Figure 9:**
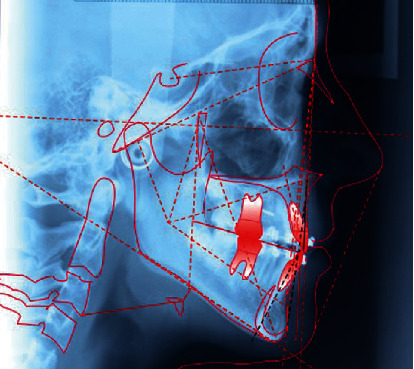
Final lateral cephalogram.

**Figure 10:**
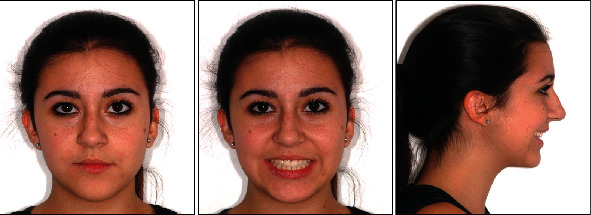
Facial photographs two years after orthodontic treatment.

**Figure 11:**
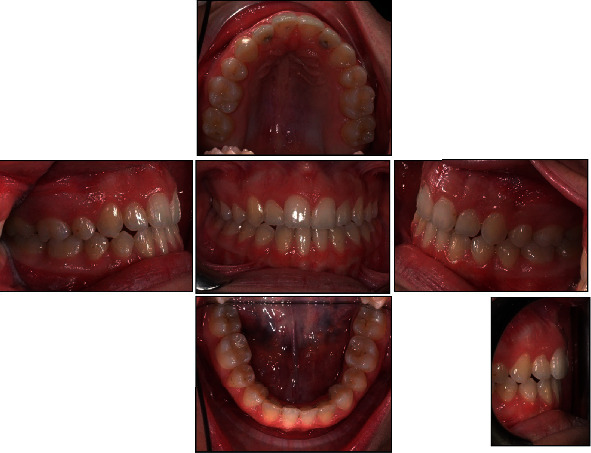
Intraoral photographs two years after orthodontic treatment.

The final intraoral and extraoral photographs are presented in Figures 7 and 8. Cephalometric tracing and values after treatment are shown in Figure 9 and Table 2. The major objectives of the treatment were achieved, such as molar class I relationship, adequate intercuspation, and tight contact points. Maxillary canines were in a class I relationship relative to the lower first premolars that were substituted for canines. The maxillary dental midline was coincident with the facial midline and lower dental midline. We accepted the overbite and overjet reduction as orthodontic compromise, due to canine substitution and to the Bolton discrepancy. After the end of the treatment, the patient received a mandibular fixed retainer and an Essix retainer. A posttreatment follow-up was carried out at 2 years; the results achieved were maintained, the teeth were well aligned, and the occlusion remained stable (Figures 10 and 11).

## 3. Discussion

There are several problems arising when hypodontia of mandibular incisors occurs bilaterally and the chosen treatment plan includes space closure by protraction of the posterior teeth and canine substitution of missing lateral incisors. In this treatment strategy, the first premolars are substituted for canines and a canine-protected occlusion becomes a premolar-protected occlusion during the mandibular lateral or working excursions [18, 19]. From an anatomic point of view, it has been reported that the first mandibular premolar frequently resembles the canine in every aspect important to substitution, such as length of the crown and root, buccal cusp height, and mesiodistal diameter [19]. Regarding the ability of the first premolar to withstand the occlusal loads, Guichet found that canines are the most capable of bearing horizontal stress, followed, in order, by first molars, first premolars, second premolars, and second molars [20]. In addition, Moyers reported that premolars possess periodontal proprioceptor impulses to the same degree as canines [21]. Furthermore, the small lingual cusp of the mandibular first premolar makes occlusal equilibration unnecessary; this procedure is usually necessary for maxillary premolars in order to prevent periodontal deterioration and to enhance the ability to withstand the occlusal loads [19]. According to previous reports, it can be concluded that the mandibular first premolar can act as an appropriate substitute for the canine, both functionally and aesthetically [18, 19]; our results are in agreement with this conclusion. The substitution of canine for missing lateral incisors may produce a tooth size discrepancy with a mandibular anterior excess due to the greater mesiodistal canine diameter (1 mm greater than the lateral incisor), while equality of tooth size can be found between the mandibular first premolar and canine [18, 19]. Bolton analysis can be used in order to assess the extent of tooth size discrepancy and to plan its correction; furthermore, it can be used for predetermining the function and aesthetic outcomes [22]. The interproximal reduction of the mandibular teeth may be recommended for achieving adequate interdigitation and ideal overjet and overbite at the end of the orthodontic treatment [18, 23–25]. The forward movement of canines for space closure may expose them to the risks of periodontal complications because of the discrepancy between the width of the alveolar bone and the size of the canine root. Moreover, an early mesial shift of the canines in the incisor area can be usually found, resulting in a well-developed alveolar bone structure [23, 26]. According to the literature, it should be pointed out that space closure by protraction of the posterior teeth and canine substitution of congenitally missing lateral incisors is and has been considered an acceptable compromise [19]. This therapeutic choice, from an aesthetic and functional point of view, would be the most conservative treatment with a better aesthetic result. The lack of bone in such an anterior segment of the mandible [27] would probably lead to imperfections during the patient's life. In this patient, considering the young age, such treatment is therefore desirable in order to reduce the risks mentioned above and the total amount of therapeutic costs. The limitations of this study are essentially due to the fact that it is a case report. The literature on the subject is sparse and not very specific for what concerns the agenesis of the lower incisors. Specifically, the article does not have a precise structure but simply wants to show how it is possible to successfully treat a case of agenesis of two lower incisors.

## 4. Conclusions

Although cases of bilateral agenesis of lower incisors are relatively rare, the clinical management of this condition always needs a careful diagnosis and treatment planning; furthermore, every patient must be evaluated individually. From the case presented in this study, we can conclude that space closure with canine substitution of missing incisors may be considered not only an acceptable clinical compromise but also an efficient treatment approach for achieving satisfactory functional and aesthetic results.

## Figures and Tables

**Table 1:** Cephalometric values at the start of the treatment.

	Value measured	Average value ± standard deviation
Skeletal		
Facial axis (BaN^PTGn)	84.6°	90.0 ± 3.0
Facial angle (Fh^NPog)	87.2°	87.0 ± 3.0
Mandibular plane to FH (GocMe^Fh)	28.0°	26.0 ± 4.0
Lower facial height (Ans^Xi^Pm)	52.6°	45.0 ± 4.0
Mandibular arc (DC^Xi^Pm)	30.2°	26.5 ± 4.0
Cranial deflection (Fh^BaN)	30.0°	27.0 ± 3.0
Maxillary A/P position		
Convexity (A-NPog)	7.7 mm	4.0 ± 2.0
Distance A-McN	5.0 mm	1.0 ± 1.0
SNA	84.0°	82.0 ± 2.0
Anterior cranial base (CC-N)	54.7 mm	56.0 ± 3.0
Middle cranial base (Pr-PTV)	37.6 mm	39.5 ± 2.0
Mandibular A/P position		
Mandibular body length (Xi-Pm)	63.0 mm	67.5 ± 3.0
Ramus Xi position (XiCF^PTV)	13.0°	15.0 ± 3.0
SNB	76.0°	80.0 ± 2.0
A-P relationship		
ANB	8.0°	2.0 ± 2.0
Dental relationships		
IMPA	89.6°	90.0 ± 2.5
L1 position (Pog-L1_|_Fh)	6.7 mm	1.5 ± 1.5
U1 position (Ans-U1_|_Fh)	-1.8 mm	0.0 ± 0
Interincisal angle (A1^B1)	123.0°	132.0 ± 6.0
Soft tissue		
Lower lip to E-plane (LL-NTPog')	-2.2 mm	0.0 ± 2.0

**Table 2:** Cephalometric values at the end of the treatment.

	Value measured	Average value ± standard deviation
Skeletal		
Facial axis (BaN^PTGn)	84.8°	90.0 ± 3.0
Facial angle (Fh^NPog)	86.8°	87.0 ± 3.0
Mandibular plane to FH (GocMe^Fh)	30.6°	26.0 ± 4.0
Lower facial height (Ans^Xi^Pm)	52.9°	45.0 ± 4.0
Mandibular arc (DC^Xi^Pm)	34.3°	26.5 ± 4.0
Cranial deflection (Fh^BaN)	27.4°	27.0 ± 3.0
Maxillary A/P position		
Convexity (A-NPog)	2.5 mm	4.0 ± 2.0
Distance A-McN	-1.0 mm	1.0 ± 1.0
SNA	78.8°	82.0 ± 2.0
Anterior cranial base (CC-N)	57.3 mm	56.0 ± 3.0
Middle cranial base (Pr-PTV)	39.7 mm	39.5 ± 2.0
Mandibular A/P position		
Mandibular body length (Xi-Pm)	64.7 mm	67.5 ± 3.0
Ramus Xi position (XiCF^PTV)	14.1°	15.0 ± 3.0
SNB	75.5°	80.0 ± 2.0
A-P relationship		
ANB	3.3°	2.0 ± 2.0
Dental relationships		
IMPA	84.8°	90.0 ± 2.5
L1 position (Pog-L1_|_Fh)	4.5 mm	1.5 ± 1.5
U1 position (Ans-U1_|_Fh)	-6.9 mm	0.0 ± 0
Interincisal angle (A1^B1)	138.9°	132.0 ± 6.0
Soft tissue		
Lower lip to E-plane (LL-NTPog')	-5.3 mm	0.0 ± 2.0
